# Book Review

**Published:** 1902-12

**Authors:** 


**Ligaments: their Nature and Morphology.** By J. Bland-Sutton. Third Edition. Pp. viii., 112. London: H. K. Lewis. 1902.—We are glad to find that a third edition of this excellent work is now issued, as it is one of those books that all "students" (in the true sense of the word) will take a delight in reading. It is only a few years ago we favourably reviewed a former edition (*vide* Vol. XV., p. 344); our opinion on this subject remains unaltered. The only fault we have to find with the volume is that its fascinating pages are too few in number.

**Acute Dilatation of the Stomach.** By H. Campbell Thomson, M.D. Pp. 54. London: Baillière, Tindall & Cox. 1902.—This is an interesting monograph based on a paper discussed at the Royal Medical and Chirurgical Society, and giving an account of fifty cases, either collected from the literature or observed by the author. The affection appears sometimes after operations or anæsthetics, and sometimes without any assignable cause. It is marked by a sudden onset, the vomiting of large quantities of fluid, very severe general symptoms, and often ends fatally in a few days. Hilton Fagge, in this country, Albrecht, Kundrat, and others have previously written on the subject. The author takes the view that the symptoms are primarily due to a paralysis of the nerves of the stomach, the walls afterwards becoming distended by gas or fluid. The paralysed stomach presses on the duodenum, where it crosses the spinal column so as to prevent any out-flow of its contents. There seems to be no pyloric stenosis, and the upper part of the duodenum together with a variable portion of the remainder of the intestines is generally dilated. The view that obstruction is caused by the superior mesenteric vessels compressing the duodenum is shown to be untenable. As to treatment: the stomach should be emptied by the stomach tube; hypodermics of strychnine and rectal feeding are recommended; while to relieve the compression of the duodenum, it is advisable to turn the patient on the right side or into a prone position, and even, if he is strong enough, into the knee-elbow one. Saline injections, to make up for the loss of the vomited fluid, may be needed; and the prognosis is usually bad whether or no surgical treatment is attempted. The writer thinks, however, that probably only the severe cases are noticed, and that mild ones may often occur and recover.

**Obstinate Hiccough.** By L. F. B. Knuthsen, M.D. Pp. viii., 169. London: J. & A. Churchill. 1902.—The title of this book fully describes its contents. Collections of cases from the British and foreign journals, with cases quoted from various text-books on medicine and extracts from various reviews, make up a pretty large volume, well printed on the lightest of paper, and easy to read when it is necessary so to do. It forms a graduation thesis, which gained honours. Four types are described: the Inflammatory, the Irritative, the Specific, and the Neurotic; and three pages of drugs and methods which have proved successful are given at the end. To those who may be called upon to treat any of these somewhat rare cases of intractable and persistent hiccough, a perusal of this cannot fail to be of assistance. The author emphasises the necessity for a careful investigation and a knowledge of the cause of the hiccough before we can expect any permanent benefit from the very numerous modes of treatment at our disposal.

**Medical Ethics: a Guide to Professional Conduct.** By Robert Saundby, M.D. Pp. viii., 88. Bristol: John Wright & Co. 1902.—The thorny topic of medical deontology is one on which a guide is often needful. Dr. Saundby has launched forth boldly, and his views are for the most part founded upon actual cases and the decisions which have been given in the leading medical journals during the past few years. Dr. Saundby's experience in these matters is great, and no one is better qualified to speak with the force of authority. Appended to the volume are extracts from the bye-laws and regulations of the General Medical Council and Medical Corporations relating to the conduct of members of the medical profession; these are the foundation of all medical ethics, and little beyond these is needed as a guide for daily conduct. Still, "it is not sufficient to say that medical ethics may be summed up in the Golden Rule": a book like this assists much in the application of the Golden Rule to the individual case, and Dr. Saundby's views are not narrow, inasmuch as he holds that consultation is permissible between all duly qualified medical practitioners, whether they may be called consultants, general practitioners, homœopaths, or mesmerists. The book is the result of much study, and the conclusions have in many cases been arrived at after consultation with many friends of judgment and influence. We are glad to welcome this record of experience and opinion by one who is *facile princeps* master of his subject; and although we cannot expect that everyone will accept all the views expressed, the profession will feel much indebted to Dr. Saundby for his courageous attempt to give us a standard of authority and a reliable guide through a field beset with pitfalls.

**Transactions of the Medical Society of the State of New York.** Published by the Society, 1902.—This volume of 528 pages is one of the most valuable works in our Exchange list. The Anniversary address, by Dr. Henry L. Elsner, of Syracuse, "On the Value to the Physician of Modern Methods of Diagnosis," is an excellent summary of recent work, and justifies the conclusion that "*the moral force of scientific methods in medicine is the greatest factor in modern medical practice*," inasmuch as "rational medicine requires for its successful practice a scientific fundament." Ten communications are each entitled "Symposium": four of these are on the physiology, the diseases, and the surgery of the pancreas; and six on hepatic diseases. Later, we find a group of four articles on general paralysis or paresis, also called a symposium—we wonder why this word has so departed from its original meaning, "a drinking party." Medical meetings may often be convivial; but a series of dry papers is scarcely worthy of this festive appellation, especially when published in a volume of *Transactions*. We must, however, admit that the papers are for the most part of exceptional merit, and the volume is full of instruction from beginning to end.

**The Johns Hopkins Hospital Reports.** Vol. X. Nos. 3, 4, 5. Baltimore: Johns Hopkins Press. 1902.—This volume contains four very interesting papers. The first is on Hodgkin's Disease, by Dr. Dorothy M. Reed, with an account of the thorough microscopical examination of eight cases. The paper especially deals with the relation of the disease to tuberculosis; and the author concludes, we think on sufficient grounds, that Hodgkin's disease is a definite clinical and pathological entity. She also describes histological changes which she regards as specific. Dr. Futcher's paper on Diabetes Insipidus enters very fully into the subject, and contributes five cases. He divides the cases clinically into primary, and secondary or symptomatic, and shows that the old classification into hydruria, azoturia, and anazoturia is no longer tenable. He considers that cerebral syphilis is a more common cause than is generally supposed. Drs. Howard and Perkins give a valuable and thorough account of the origin and occurrence of eosinophil cells in normal and pathological tissues. And the concluding paper is one by Dr. Lynch on the placental transmission of typhoid fever, showing *inter alia* that the typhoid bacillus may pass from mother to child in utero, and set up fœtal septicæmia, the child dying in utero, or soon after birth.

**A Manual of Medical Treatment, or Clinical Therapeutics.** By I. Burney Yeo, M.D. New and Revised [Third] Edition. Two Volumes. Pp. xiii., 696; vii., 818. London: Cassell and Company Limited. 1902.—The object of this work is the study of disease from the point of view of treatment. The teaching of therapeutics is here approached from the side of the disease, and not from the side of the drug or remedy. The author points out that "it is by examining into the mode of *causation* of disease, by investigating the true nature of the morbid changes which underlie the *phenomena* of disease, and by an exact knowledge of the properties and mode of action of the agencies we introduce for the purpose of influencing favourably its course, that what are termed *rational indications* for treatment are arrived at." It is the author's aim to deduce rational indications for treatment from an examination of the pathological nature and the clinical course and character of the disease under discussion. These volumes, teeming with common-sense methods of treatment, the greater part of which are found to be more or less useful, give the lie direct to the views of the satirist, that the art of drugging consists of pouring in substances of which little is known into bodies of which we know less. Those who use only few drugs are commonly those who have only learned to use a few; and those who boast that they rarely give drugs at all must neglect many opportunities of doing things well worth doing. Medicine is not yet out of date, and operations are not everything. Rational therapeutics, grounded on physiology and pathology, must in the long run prevail over all the artifices of the empiric and the charlatan. Our previous review (Vol. XII., 1894, p. 125) states that the book is to be commended "as a useful and practical one, based on sound scientific principles, well adapted to the needs of everyday work, and supplying a distinct want in medical literature." The appearance of the volumes of the first edition taken from our Library shows that it has been distinctly popular, and that a new edition is likely to be more so. There is no great difference in the size of the new edition, the preparation of which has been no light task; it has been needful to execute a most careful sifting in order to retain what is likely to be of lasting service, and to discard that which, at best, can only be destined to a temporary popularity. Numerous additions have been made, such as Hay Fever, Paralysis Agitans, Cerebral Tumours, Erysipelas, Cerebro-Spinal Fever, Rickets, Scurvy, and Purpura. We can only congratulate the author on the efficiency and utility of his work and on the fact that the most recent introductions have not been overlooked; for the very complete chapters on the climatic and medicinal treatment of phthisis conclude with a list, extending to nearly five pages, of sanatoria for the reception of paying patients. A general index of nearly fifty pages gives the reader every opportunity of finding the latest information on almost every variety of medical treatment.

**Clinical Methods.** By ROBERT HUTCHISON, M.D., and HARRY RAINY. New and Enlarged [Second] Edition. Pp. xii., 612. London: Cassell & Company Limited. 1902.—Clearness, conciseness, and accuracy are essential characteristics for any handbook on practical clinical work, and we have no hesitation in saying that these are the features of this handy manual, which will be found of very great service to students and practitioners alike. It is very complete: the authors have not allowed themselves to stray outside the proper scope of their work, and they have given throughout the book detailed instructions and practical hints which could only occur to those who are constantly in touch with students. We know of no better guide to place in the hands of those entering on work in the medical wards of a hospital, or for practitioners who desire to keep themselves well versed in the more recent developments of practical medicine.

**Gibson and Russell's Physical Diagnosis.** Third Edition, revised and rewritten by FRANCIS D. BOYD, C.M.G., M.D. Pp. xvii., 448. Edinburgh: Young J. Pentland. 1902.—The third edition of this work has been largely revised and re-written. The new sections deal with examination of the blood; examination of gastric contents; intestinal parasites; cranial nerves; and clinical bacteriology. The information given under each of these heads is essentially practical and thoroughly up to date. Of especial value is the chapter on the chemical examination of stomach contents, and the interpretation of the results obtained, but the classification adopted of the white blood cells is one which is likely to lead to confusion, and the descriptions of some of the cells, such as the myelocyte, is meagre and unsatisfactory. The book as a whole is one which can be most heartily recommended as an aid both to students and practitioners.

**A Manual of Surgery.** By WILLIAM ROSE, M.B., B.S., and ALBERT CARLESS, M.S. Fifth Edition. Pp. xiv., 1213. London: Baillière, Tindall & Cox. 1902.—The production of five editions of a book in four years is sufficient to show that the work has filled a definite want. The authors have wisely resisted the tendency to enlargement which so often accompanies new editions, and which thereby destroys much of their *raison d'être*. The present arrangement is sound and practical, and the volume is certainly one of the best of the smaller manuals of surgery. Some of the errors of the first edition, however, still exist. In speaking of the diagnosis of calculi by X-rays, it is stated that "phosphatic calculi are more easily detected than those consisting of oxalate of lime," and nephrorrhaphy is defined as "the title given to the operation for fixing a movable kidney." The book is not well bound.

**Hydatid Disease of the Lungs.** By ALFRED AUSTIN LENDON, M.D. Pp. viii., 122. London: Baillière, Tindall & Cox. 1902. —The author, since leaving Bristol to practise in Adelaide, has had ample opportunity of seeing and treating a large number of cases of hydatid disease, as this disease is so much more frequently met with in South Australia than in this country. These clinical lectures are a most useful addition to the literature of the subject referred to, and they are placed before the reader in a clear, practical manner; the cases quoted are a well-selected series, and numerous valuable hints are given with regard to the diagnosis and treatment of the individual cases. It will be wise for surgeons here to bear in mind the conclusions of the author concerning the invasion of the thorax by cysts of abdominal origin. They are these: "Short, however, of certainty, I should lay great stress (1) on the possibility of any hydatid at the base of the right thorax being hepatic in origin; (2) on the probability of any case in which a daughter cyst has been expectorated turning out to be hepatic, because daughter cysts are comparatively uncommon in hydatid disease of the lungs." The text is well illustrated, and such details as printing and paper have received so much consideration that the result is excellent. In any future edition we would suggest that the last chapters be so arranged as to come earlier in the volume.

**An Introduction to Dermatology.** By NORMAN WALKER, M.D. Pp. xvi., 301. Bristol: John Wright & Co. 1902.—We had great satisfaction in reviewing this work about three years ago. It still retains its modest title; and the fact that another edition is called for under three years speaks for itself. The new edition is larger, as might be expected, containing 301 pages as against 247 in the first. The bulk of the new matter is added to bring the contents up to date, and there is besides a considerable increase in new plates, printed upon better paper, and also more illustrations of the text. We notice, with much appreciation, that the author admits the description of Herpes Zoster, or "Zoster" as it is often termed, as an "acute disease of the nervous system"; and he introduces some of Head's interesting diagrams. We were well pleased with the chapters on Eczema in the first issue, and in the present volume they are still more interesting. No doubt many of the so-called varieties of eczema will, as time goes on and knowledge increases, be relegated to their proper places, and the complications of eczema be correspondingly reduced. Some very important observations concerning seborrhœa and acne are recorded; but we cannot agree with the author that psoriasis should be classed with seborrhœa, the two present such important differences, and amongst them the comparative rareness of psoriasis of the scalp, or its insignificance generally when the scalp is affected, are not points to be lightly set aside. The book is fully indexed, and is one to be appreciated by students and busy practitioners.

**The Treatment of Neurasthenia.** By A. PROUST and GILBERT BALLET. Translated by PETER CAMPBELL SMITH. Pp. xiv., 213. London: Henry Kimpton. 1902.—This will be found a useful book for the medical practitioner; in it he will find a good account of the varieties of neurasthenia, and of the treatment to be adopted. In present-day practice, an increasing number of patients suffer from this disease largely as a consequence of the wear and tear of the conditions of modern life; and it is essential in treatment to have a thorough knowledge of the disease, as a fundamental point for success in treatment is to be able to enter into a sympathetic appreciation of the patient's sufferings. A great deal can be done for them by an intelligently conceived line of treatment, and from this book many useful hints in this direction may be gained.

**Aids to Practical Dispensing.** By C. J. S. THOMPSON. Third Edition, Enlarged. Pp. viii., 92. London: Baillière, Tindall & Cox. 1902.—This little book deals in a very comprehensive manner with the many practical difficulties of dispensing. It is doubtful whether pharmacy can ever be learnt from the pages of a book; but as an adjunct to the dispensing counter, any student, medical or pharmaceutical, will find it useful. Giving the first chapter to general principles and manipulation of apparatus, it deals in successive chapters with the various forms of the problems set by the prescriber, not only with the mixtures, emulsions, pills, plasters, and suppositories of an older day, but including a chapter on the compressed tablets and pastilles that characterise the 20th century prescription. The paragraphs on incompatibles and pill excipients will be found very useful. Under "weighing and measuring" in chapter one, some reference might with advantage have been made to that great difficulty of the average student, the relation between weight and measure, and its application to percentage solutions prepared by English weights. Regulations for the keeping and selling of poisons, a table of solubilities, and a glossary of Latin terms—some of which, by the way, need considerable revision—complete a very useful little manual.

**Merck's Index.** 11 Auflage—Abgeschlossen Ende Juli, 1902. Darmstadt: Eduard Roether.—This volume of 374 double pages is the second edition of the index published at Darmstadt in 1897. During the five years, ten thousand copies have been distributed. The original plan of the work has been followed, chief consideration having been given to the needs of physicians and pharmacists. The index is divided into six parts: the first comprises chemical bodies and definite principles, certain Galenical preparations and organic extracts; the second, special solutions and forms of substances used in analytical and microscopical work; the third, crude drugs; the fourth, minerals; the fifth, collections suitable to schools and museums; and the sixth, sundries. Throughout the various sections brevity and compactness have been aimed at; and to aid in this object abbreviations have been used, of which full explanations are given. In the therapeutic notes special attention has been given to dosage; the indications for the uses of remedies have been carefully revised, and in treating of poisonous bodies their antidotes have been noted.

**The Pharmacopœia of the Hospital for Diseases of the Throat, Nose, and Ear.** Edited by H. Lambert Lack, M.D., and Charles A. Parker. Sixth Edition. Pp. ix., 75. London: J. & A. Churchill. 1901.—This eminently useful epitome of formulæ for use in diseases of the throat, nose, and ear is brought well up to date. The pharmacopœia has been improved in several respects, especially in the addition of a series of formulæ from the most recent local anæsthetic preparations, and in the metrical system equivalents of the ingredients being appended to the British Apothecaries' weights and measures.

**Christian Science, Medicine, and Occultism.** By Albert Moll, M.D. Translated from the German by F. J. Rebman. Pp. 47. London: Rebman Limited. 1902.—Dr. Moll is a careful investigator of hypnotism, and after reading his book on this subject in the "Contemporary Science Series," one is a little surprised that he should take the trouble to seriously criticise so-called "Christian Science." He does this, however, in a fair, impartial manner, and in a scientific spirit. He looks upon Christian Science as a psychical epidemic, spreading along "lines of communication" like other infectious complaints, and, like them, difficult to check. Charlatanism is, however, more easily dealt with in Germany than in England, for we are told that a few years ago S—— was sent to prison for treating people with prayer and footbaths, etc. "For this performance S—— received from each of his patients eight shillings, and from the police magistrate one month." The extreme cleverness evinced by Mrs. Eddy in avoiding rational conclusions as to the effect of poison, etc., is very entertaining; and she is certainly humorous when she points out that "particular care should be bestowed upon patients who have been taking physic for some time, *and in consequence are run down*." Dr. Moll (pp. 29 and 30) is rather too severe on specialists. He "hopes that this movement will eventually succeed in suppressing specialism, and re-establishing the general practitioner in that position to which he has a well-founded claim." He very justly notices that these people (*i.e.*, the followers of Christian Science, etc.) are easily impressed by anything which deviates from the normal rule; "thus it may not be a mere accident . . . that many spiritualists are strong believers in Dr. Jaeger's sanitary wool theory, are enthusiastic homœopaths, or strict vegetarians, are severe enemies of the regular school of medicine, and—who can tell?—perhaps also inveterate Baconites."

**The Mattison Method in Morphinism.** By J. B. MATTISON, M.D. Pp. 40. New York: E. B. Treat & Company. 1902.—This monograph is the outcome of thirty years' experience in the study and treatment of this disease. The method commended is a mean between two extremes—"avoiding the painful ordeal of abrupt disuse, and the tiresome delay of prolonged decrease,—and is based on the power of certain remedial resources to subdue abnormal reflex action, and secures, largely, two cardinal objects—minimum duration of treatment, and maximum freedom from pain." The drug which is to do all this is bromide; the initial dose of the sodium salt is ten grains, twice daily, the dose being daily increased up to a maximum of 100 grains twice in twenty-four hours. "Each case is a law unto itself; and the length and amount of the bromide giving, and consequent rate of opiate decrease, is determined entirely by individual peculiarity." Two other drugs, codein and trional, have their uses, and form with the bromides the main remedies on which reliance is placed. The book abundantly proves that morphinism is vincible.

